# Feruloylation and structure of arabinoxylan in wheat endosperm cell walls from RNAi lines with suppression of genes responsible for backbone synthesis and decoration

**DOI:** 10.1111/pbi.12727

**Published:** 2017-04-21

**Authors:** Jackie Freeman, Jane L. Ward, Ondrej Kosik, Alison Lovegrove, Mark D. Wilkinson, Peter R. Shewry, Rowan A.C. Mitchell

**Affiliations:** ^1^ Plant Biology and Crop Science Rothamsted Research Harpenden Hertfordshire UK

**Keywords:** hydroxycinnamic acids, xylan acylation, grass xylan, wheat endosperm cell wall

## Abstract

Arabinoxylan (AX) is the major component of the cell walls of wheat grain (70% in starchy endosperm), is an important determinant of end‐use qualities affecting food processing, use for animal feed and distilling and is a major source of dietary fibre in the human diet. AX is a heterogeneous polysaccharide composed of fractions which can be sequentially extracted by water (WE‐AX), then xylanase action (XE‐AX) leaving an unextractable (XU‐AX) fraction. We determined arabinosylation and feruloylation of AX in these fractions in both wild‐type wheat and RNAi lines with decreased AX content (TaGT43_2 RNAi, TaGT47_2 RNAi) or decreased arabinose 3‐linked to mono‐substituted xylose (TaXAT1 RNAi). We show that these fractions are characterized by the degree of feruloylation of AX, <5, 5–7 and 13–19 mg bound ferulate (g^−1^ AX), and their content of diferulates (diFA), <0.3, 1–1.7 and 4–5 mg (g^−1^ AX), for the WE, XE and XU fractions, respectively, in all RNAi lines and their control lines. The amount of AX and its degree of arabinosylation and feruloylation were less affected by RNAi transgenes in the XE‐AX fraction than in the WE‐AX fraction and largely unaffected in the XU‐AX fraction. As the majority of diFA is associated with the XU‐AX fraction, there was only a small effect (TaGT43_2 RNAi, TaGT47_2 RNAi) or no effect (TaXAT1 RNAi) on total diFA content. Our results are compatible with a model where, to maintain cell wall function, diFA is maintained at stable levels when other AX properties are altered.

## Introduction

The composition of the cell walls of wheat grain is unusual in that they are largely comprised of arabinoxylan (AX), with no lignin and low amounts of cellulose in starchy endosperm and aleurone tissues. Starchy endosperm cell walls are primary cell walls containing 70% AX, 20% β‐(1,3;1,4) glucan, 2%–7% glucomannan and 2%–4% cellulose. The composition of aleurone cell walls is similar to that of starchy endosperm tissue comprising 65% AX, 30% β‐(1,3;1,4) glucan and 2% glucomannan and cellulose, whereas, whilst AX is still the major component of pericarp cell walls (60% AX), they also contain 30% cellulose and 12% lignin (Shewry *et al*., 2014). AX consists of a linear backbone of (1→4)‐linked xylopyranose (Xyl*p*) residues which may be mono‐substituted with α‐(1,3) linked arabinofuranose (Ara*f*) or di‐substituted with both α‐(1,2) and α‐(1,3) linked Ara*f*. The hydroxycinnamic acids ferulic acid (FA) and *p*‐coumaric acid (*p*CA) can occur ester‐linked to the mono‐substituted α‐(1,3) linked Ara*f*. Ester‐linked *p*CA is not detected in pure starchy endosperm tissue dissected from wheat grain but is concentrated in the aleurone layer of wheat grain (Barron *et al*., 2007). Ester‐linked ferulic acid can undergo oxidative dimerization forming ether or C–C bonds linking chains of AX or glucuronoarabinoxylan (GAX) (Ishii, 1991), or of (G)AX to lignin in lignified tissues (Ralph *et al*., 1995).

The structure and solubility of wheat cell walls have important effects on a number of end users including consumption by humans and animals, and alcohol and biofuel production. The cell walls also provide mechanical strength and protection against pathogen attack. As the major component of wheat grain cell walls, the structure and properties of AX are of great importance. The solubility of AX molecules is affected by the degree of Ara*f* substitution and the distribution pattern of Ara*f* residues on the xylan backbone (Hoije *et al*., 2008): Ara*f* substitution hinders hydrogen bonding between xylan chains and favours solubility of the polymer. The degree of feruloylation also affects the solubility of AX. The formation of the covalent diferulate cross‐links between AX molecules occurs via oxidative coupling using free radicals, probably generated by peroxidases (Ralph, 2010). The greater the degree of feruloylation the more cross‐linking is likely to occur, decreasing the solubility of AX. In starchy endosperm cell walls, the degree of feruloylation is much lower than in other tissues of the grain being fivefold less per unit Xyl*p* than in outer tissues (Barron *et al*., 2007; Saulnier *et al*., 2007). However, as starchy endosperm cell walls have little cellulose and no lignin, it is possible that, despite the low amount, feruloylation of AX is critical in maintaining the structural integrity of starchy endosperm cell walls; heterologous expression of a feruloyl esterase in starchy endosperm resulted in endosperm collapse in some transgenic wheat lines (Harholt *et al*., 2010). There is also some variation in AX structure between different parts of the endosperm (Saulnier *et al*., 2009; Toole *et al*., 2010). These differences may reflect a requirement for different cell wall properties in different parts of the starchy endosperm.

Arabidopsis genes encoding IRX9 and IRX14, in the glycosyl transferase (GT) 43 family, and IRX10, in the GT47 family, were identified in genetic screens as being essential for normal extension of the xylan backbone (Brown *et al*., 2007, 2009; Pena *et al*., 2007). Subsequent studies demonstrated that the IRX10 proteins can extend xylan chains *in vitro* without IRX9 or IRX14; therefore, it is likely that IRX9 and IRX14 are accessory proteins required for xylan extension *in planta* and that IRX10 is the catalytic unit directly responsible for extension of the xylan chain (Jensen *et al*., 2014; Urbanowicz *et al*., 2014). Recent evidence supports the concept that IRX9, IRX14 and IRX10 participate in a xylan synthase complex (Zeng *et al*., 2016).

Based on greater expression in grasses than in dicots, we previously identified candidate genes for AX biosynthesis in the GT43, GT47, GT61 gene families and for feruloylation of AX in a grass‐specific clade of the BAHD acyl‐coA transferase superfamily (Mitchell *et al*., 2007). Close homologues of IRX9, IRX14 and IRX10 are highly expressed throughout wheat starchy endosperm development (Pellny *et al*., 2012). We have shown that in transgenic wheat lines with RNAi constructs targeting suppression of the most highly expressed homologues of *IRX9* (*TaGT43_2*) and *IRX10* (*TaGT47_2*) in starchy endosperm, AX amount is decreased by up to 50% (Lovegrove *et al*., 2013). Amongst GT gene families, the greatest expression bias in favour of grasses compared to dicots was in the GT61 gene family (Mitchell *et al*., 2007). We also showed that wheat and rice genes (*XAT1*,* 2* and *3*) in the GT61 family are responsible for addition of α‐(1,3) linked Ara*f* to xylan; RNAi suppression in wheat starchy endosperm of the most highly expressed GT61 gene (*TaXAT1*) results in a 70%–80% reduction in α‐(1,3) Ara*f* substitution of mono‐substituted Xyl*p* in AX (Anders *et al*., 2012). Three independent transgenic wheat lines per target, in which expression of the *TaGT43_2*,* TaGT47_2* or *TaXAT1* genes was suppressed in starchy endosperm, were used to study the effects on chain length of water‐soluble AX (WE‐AX) and AX solubilized by alkaline extraction (AE‐AX) and on extract viscosity (Freeman *et al*., 2016). Suppression of *TaGT43_2* and *TaGT47_2* genes resulted in decreased AX chain length in both fractions and a decrease in extract viscosity of up to sixfold whereas suppression of *TaXAT1* resulted in a population shift towards shorter chain length in WE‐AX with little effect on AE‐AX and a more modest decrease in extract viscosity by twofold.

Feruloylation of AX is a key property of grass cell walls, allowing cross‐linking which confers structural strength and determines digestibility (de Oliveira *et al*., 2015), and ferulate content of wheat grain is important with respect to diet because it may confer health benefits (Shewry and Hey, 2015). We have therefore determined whether AX feruloylation and ferulate dimerization were affected in the wheat RNAi lines with radically altered AX structure. To this end, we examined feruloylation and arabinosylation in different fractions extracted from wheat white flour (which is essentially equivalent to starchy endosperm tissue) in these RNAi lines. Using enzymatic digestion to solubilize water‐unextractable AX without removing ester‐linked FA, we determined the distribution of AX between different fractions (water‐extractable, xylanase‐extractable and xylanase‐unextractable), the nature of arabinosylation and the degree of feruloylation and dimerization of ferulate in these fractions in the TaGT43_2, TaGT47_2 and TaXAT1 RNAi lines.

## Results

### Effect of suppression of AX biosynthetic genes on feruloylation and structure of AX in white flour

We have previously demonstrated that transgenic RNAi lines suppressing *TaGT43_2* and *TaGT47_2* genes encoding components of xylan synthase have 40%–50% decreases in total AX, whereas RNAi lines suppressing *TaXAT1* which encodes an arabinosyl transferase have only 0%–15% decreases (Anders *et al*., 2012). The abundance of AX oligosaccharides (AXOS) containing xylose mono‐ and di‐substituted by Ara*f* was measured in solubilized fractions and mono‐ and di‐substituted AXOS were shown to be similarly decreased in TaGT43_2 and TaGT47_2 RNAi lines (Lovegrove *et al*., 2013), whereas in TaXAT1 RNAi lines, there was no effect on di‐substituted but a 70‐80% decrease in mono‐substituted AXOS (Anders *et al*., 2012). However, the differences in FA which occurs ester‐linked to a proportion of these 3‐linked Araf have not been previously reported. The bound FA and FA dimer contents of multiple transgenic lines are summarized in Table 1. It is assumed that all bound ferulate in wheat endosperm is ester‐linked to AX (Saulnier *et al*., 2007) so an overall value of FA and diFA per unit AX where AX is estimated from monosaccharide is also given. There is a decrease in amount of FA monomer in all TaGT43_2 and TaGT47_2 lines and in two of the three TaXAT1 RNAi lines per unit dry weight (dwt), but per unit AX, there was no consistent effect of the transgenes. FA dimer was less affected than monomer by transgenes per unit dwt and was consistently increased per unit AX. We decided to look at these changes in AX structure in selected representative lines in more detail by examining different fractions of AX to provide insight into the properties of endosperm AX.

**Table 1:** Bound ferulic acid monomer (FA) and dehydrodimer (diFA) content of white flour from transgenic wheat lines homozygous (H) for RNAi constructs suppressing AX biosynthetic genes, and azygous (A) control null segregant lines. Experiment number (Expt.) indicates which lines were grown together in glasshouse experiments. Expt. 1 was a randomized block design (*n* = 4), Expt. 2 and Expt. 4 were randomized design (*n* = 4) and Expt. 3 did not have biological replication. Contents are expressed per unit dry weight flour and per unit arabinoxylan (AX). Ratio of H/A is shown as mean % ± SE (calculated using variance of H and A) with *P*‐value for H‐A comparison in parentheses

Expt.	Line	Trans‐gene	FA μg/g d.wt.		diFA μg/g d.wt.		FA μg/g AX		diFA μg/g AX	
			Mean ± SE	H/A % (*P*‐value)	Mean ± SE	H/A % (*P*‐value)	Mean ± SE	H/A % (*P*‐value)	Mean ± SE	H/A % (*P*‐value)
1	TaGT43_2‐3	A	94 ± 2	69 ± 3 (<0.001)	19.5 ± 0.4	82 ± 2 (0.008)	4464 ± 75	126 ± 5 (0.007)	927 ± 19	149 ± 3 (<0.001)
		H	65 ± 2		16.0 ± 0.3		5637 ± 179		1385 ± 11	
1	TaGT43_2‐6	A	111 ± 5	71 ± 4 (<0.001)	20.5 ± 0.9	90 ± 5 (0.085)	5663 ± 380	88 ± 6 (0.085)	1043 ± 65	110 ± 8 (<0.001)
		H	79 ± 2		18.4 ± 0.8		4958 ± 142		1147 ± 34	
2	TaGT43_2‐5	A	161 ± 2	45 ± 5 (<0.001)	29.0 ± 5.7	82 ± 18 (0.436)	6855 ± 403	85 ± 9 (0.169)	1190 ± 199	162 ± 31 (0.031)
		H	72 ± 8		23.8 ± 2.4		5794 ± 548		1921 ± 169	
1	TaGT47_2‐1	A	81 ± 4	78 ± 5 (0.003)	17.1 ± 1.1	86 ± 7 (0.061)	3812 ± 381	145 ± 16 (<0.001)	807 ± 94	159 ± 20 (0.21)
		H	64 ± 2		14.8 ± 0.6		5523 ± 257		1282 ± 50	
1	TaGT47_2‐4	A	117 ± 6	59 ± 4 (<0.001)	25.2 ± 1.2	74 ± 5 (<0.001)	5968 ± 325	92 ± 6 (0.218)	1290 ± 63	116 ± 7 (0.014)
		H	68 ± 2		18.6 ± 0.8		5514 ± 135		1501 ± 54	
3	TaGT47_2‐7	A	133	67	30.5	80	5537	99	1272	120
		H	89		24.3		5503		1520	
4	TaXAT1‐1^a^	A	123 ± 8	86 ± 8 (0.071)	18.1 ± 1.0	87 ± 6 (0.006)	5606 ± 353	99 ± 9 (0.906)	821 ± 43	100 ± 6 (0.994)
		H	106 ± 8		15.8 ± 0.6		5540 ± 398		820 ± 30	
4	TaXAT1‐2	A	99 ± 6	101 ± 10 (0.922)	14.9 ± 0.4	107 ± 5 (0.179)	4798 ± 422	139 ± 15 (0.004)	721 ± 40	148 ± 9 (<0.001)
		H	100 ± 8		16.0 ± 0.6		6652 ± 456		1066 ± 29	
4	TaXAT1‐3	A	117 ± 1	73 ± 4 (0.002)	15.5 ± 0.2	93 ± 3 (0.167)	5574 ± 140	102 ± 9 (0.885)	743 ± 15	130 ± 8 (<0.001)
		H	85 ± 5		14.5 ± 0.5		5656 ± 506		964 ± 59	

a No biological replication of AX content. AX content is taken from Freeman *et al*. (2016).

### Composition of fractions sequentially extracted from wild‐type wheat white flour

**Figure 1:**
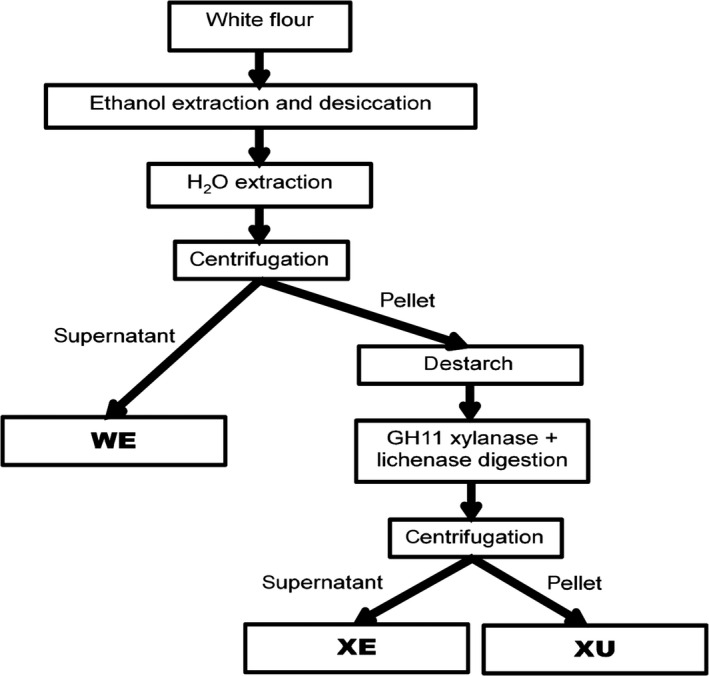
Scheme representing the method for sequential extraction of fractions containing arabinoxylan with different properties from alcohol insoluble residue from wheat white flour. WE = water‐extractable fraction; XE = xylanase‐extractable fraction solubilized by digestion with GH11 endoxylanse and lichenase; XU = xylanase‐unextractable fraction.

White flour was separated into three fractions: a water‐extractable fraction (WE); a fraction released by digestion with endoxylanse GH11 and lichenase (XE) and the remaining fraction which was insoluble in water and not solubilized by digestion (XU) (Figure 1). The composition of total white flour and of the different fractions isolated from white flour of wheat cv. Cadenza was analysed (Table 2). All galactose present in the WE fraction is assumed to be from arabinogalactan (AGP). Glucose and mannose in the XU fraction are most likely from glucomannan. WE‐AX content is consistent with amounts reported by (Gebruers *et al*., 2010) for Cadenza grown at multiple sites and years (4.3–6.9 mg/g dry weight), but TOT‐AX content is less than reported by these authors (23.5–27.3 mg/g dry weight). The sum of AX content measured in the three fractions (18.4 mg/g dry weight) is similar to that measured in the unfractionated white flour (18.7 mg/g dry weight) indicating good recovery of AX during extraction. As expected, the majority of the AX is in the XE fraction (64%) and more than 90% of the AX is extracted with only 8% in the XU fraction (28% in the WE fraction). The A/X ratio is lowest in the WE fraction, slightly but significantly greater in the XE fraction (*P *=* *0.012), but in the XU fraction, there is approximately three times the amount of arabinosylation as in the WE and XE fractions. The A/X ratios of the WE and XE fractions are consistent with those reported for twenty wheat cultivars by (Ordaz‐Ortiz and Saulnier, 2005).

**Table 2:** Composition of fractions sequentially extracted from wheat white flour cv Cadenza. Sugar contents are in mg/g dry weight flour, bound ferulic acid monomer and total dehydrodimers contents are in μg/g dry weight flour (d.wt.) or μg/g arabinoxylan (AX) from the same fraction. WE = water‐extractable fraction; XE = xylanase‐extractable fraction solubilized by digestion with GH11 endoxylanse and lichenase; XU = xylanase‐unextractable fraction; TOT = total without fractionation. Values are average ± SEM, *n* = 4 replicate extractions except * where *n* = 2

	Fraction			
	WE	XE	XU	TOT
Arabinose	2.9 ± 0.02	4.3 ± 0.08	1.0 ± 0.06	10.0 ± 0.18
Xylose	3.4 ± 0.03	7.5 ± 0.15	0.6 ± 0.05	11.6 ± 0.28
Galactose	1.6 ± 0.01	Trace	0.3 ± 0.03	4.2 ± 0.07
Glucose	6.4*	8.6 ± 0.28	3.0 ± 0.05	728.1 ± 11.04
Mannose	Trace	Trace	0.6 ± 0.04	1.2 ± 0.14
AX^b^	5.2 ± 0.02	11.7 ± 0.22	1.5 ± 0.10	18.7 ± 0.40
A/X	0.52 ± 0.009	0.57 ± 0.009	1.65 ± 0.091	0.86 ± 0.011
Ferulic acid (μg/g d.wt.)	12.0 ± 0.44	54.2 ± 2.81	36.6 ± 0.58	103.4 ± 1.19
Diferulic acid^a^ (μg/g d.wt.)	1.0 ± 0.07	8.1 ± 0.45	8.71 ± 0.17	21.0 ± 0.59
Ferulic acid (μg/g AX)	2313 ± 92	4634 ± 281	24022 ± 1373	5540 ± 64
Diferulic acid^a^ (μg/g AX)	191 ± 12	688 ± 41	5705 ± 308	1131 ± 31
Percentage of bound ferulic acid as dimer	8%	13%	19%	17%

a sum of amounts of 8‐5′ benzofuran, 8‐O‐4′, 5‐5′ and 8‐5′ diferulates. Three further diferulate standards (8‐8′, 8‐8′ aryltetralin and 8‐8′ tetrahydrofuran) were not detected in these samples.

b Sum of arabinose and xylose corrected for arabinogalactan content.

The degree of feruloylation of AX varied widely between the different AX fractions. Despite only accounting for 8% of the total AX content, the fraction which was not solubilized by enzymatic digestion (XU) contained 36% of the bound ferulate monomer and 49% of the dimers meaning that the AX in this fraction had 10 times the amount of bound FA monomer and 30 times the amount of dimer than in the WE‐AX fraction (Table 2). The per cent dimerization of bound FA was 7.6%, 12.9% and 19.2% in the WE, XE and XU fractions, respectively. The values for FA and FA dimers are similar to those previously reported for WE and water‐unextractable fractions (equivalent to our XE + XU_fractions combined) of wheat flour AX (Dervilly‐Pinel *et al*., 2001).

### Effect of suppression of AX biosynthetic genes on the distribution of AX between the fractions and the structure of AX in the fractions

**Figure 2:**
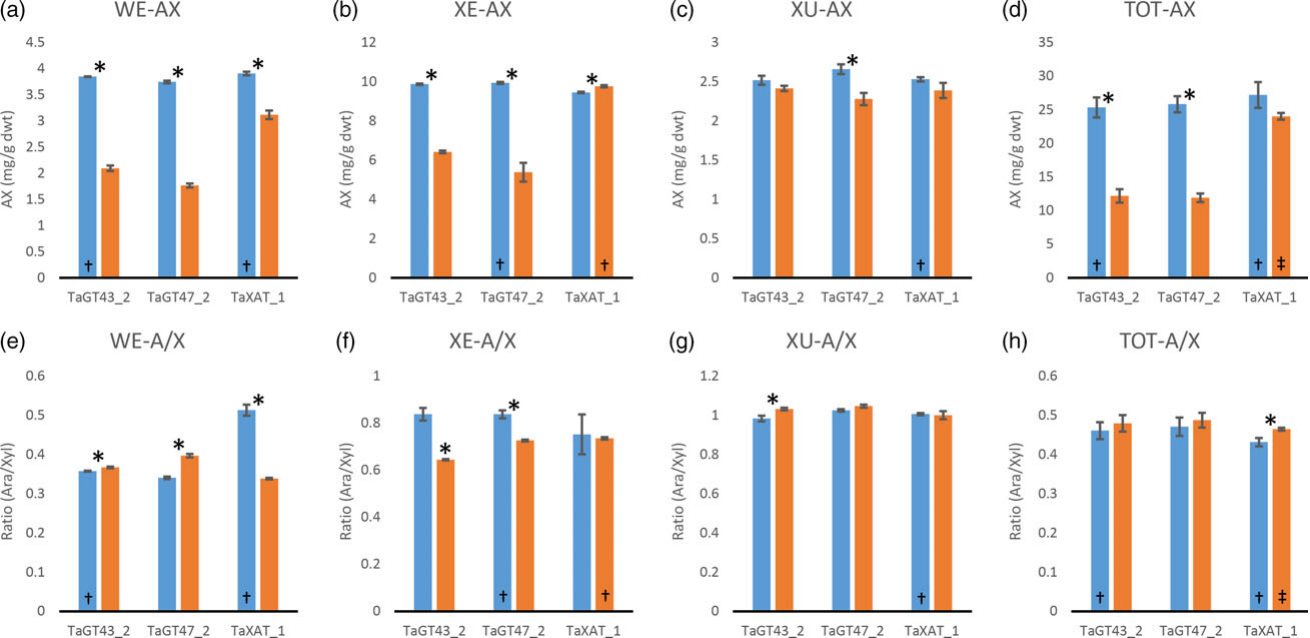
a to d: arabinoxylan content of white flour (TOT‐AX) and fractions sequentially extracted from white flour from transgenic wheat lines homozygous for RNAi constructs suppressing AX biosynthetic genes (orange bars), and azygous control lines (blue bars). e to h: ratio of arabinose to xylose in AX in the same samples as in a to d. Values are average ± SEM,* n* = 4 replicate extractions except bars marked † where *n* = 3 and ‡ where *n* = 2; asterisks denote significant differences at *P* < 0.05 from Student's *t*‐test.

**Figure 3:**
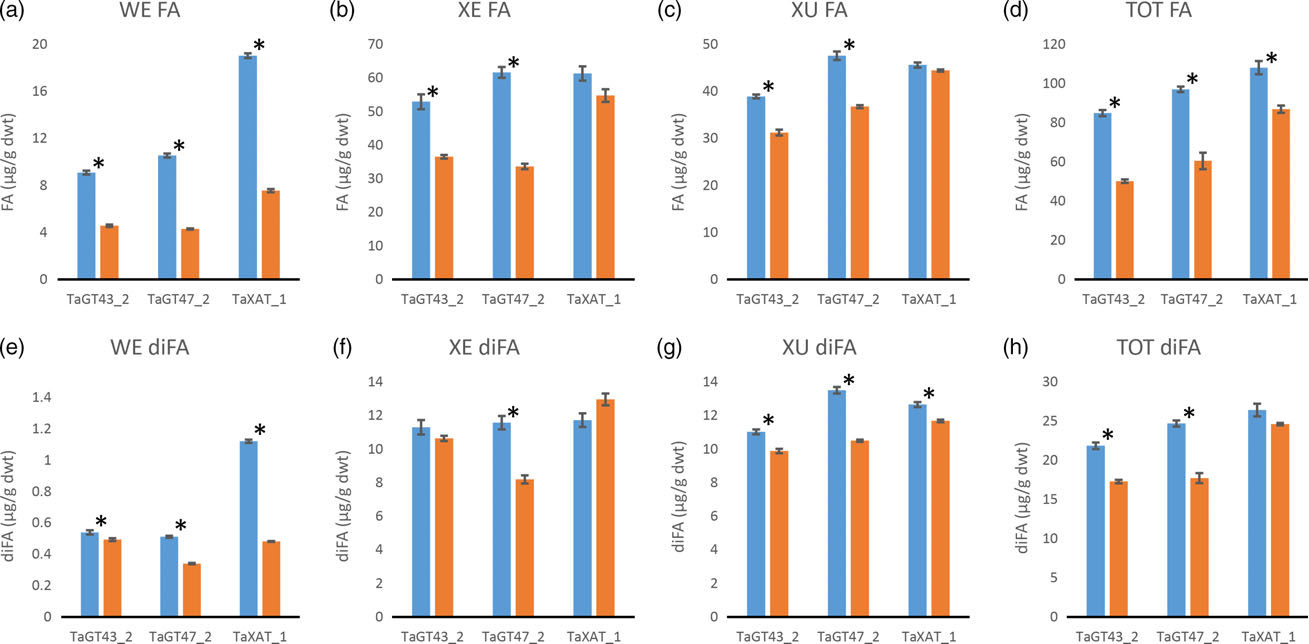
a to d: Bound ferulic acid monomer (FA) content of white flour (TOT‐AX) and fractions sequentially extracted from white flour from transgenic wheat lines homozygous for RNAi constructs suppressing AX biosynthetic genes (orange bars), and azygous control lines (blue bars). e to h: Bound ferulic acid dehydrodimer (diFA) content in the same samples as in a to d. Dimers are the sum of amounts of 8‐5′ benzofuran, 8‐O‐4′, 5‐5′ and 8‐5′ diferulates (separate values given in Table S2). Values are average ± SEM; for WE, XE and XU *n* = 4 replicate sequential extractions for TOT *n* = 3; asterisks denote significant differences at *P* < 0.05 from Student's *t*‐test.

**Figure 4:**
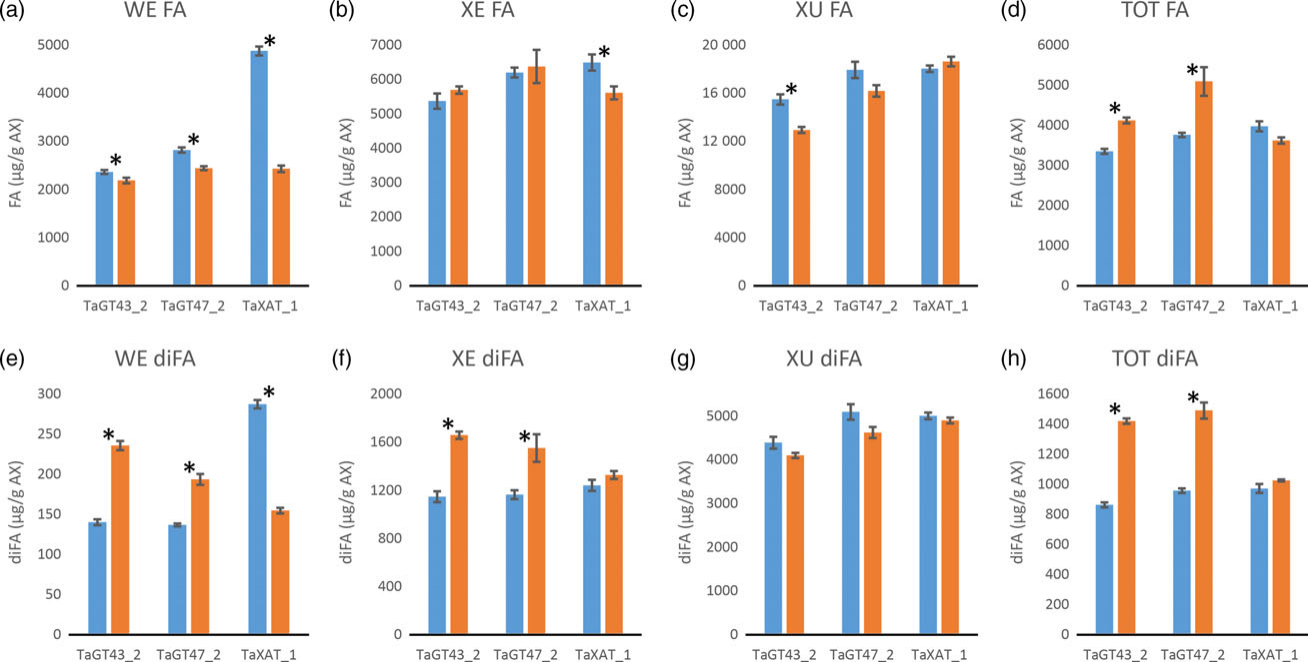
Bound ferulic acid monomer (FA) (a‐d) and dimer content (diFA) (e‐h) per unit of arabinoxylan (AX) in white flour (TOT‐AX) and fractions sequentially extracted from white flour from transgenic wheat lines homozygous for RNAi constructs suppressing AX biosynthetic genes (orange bars), and azygous control lines (blue bars). Values are average ± SEM; for WE, XE and XU *n* = 4 replicate sequential extractions for TOT *n* = 3; asterisks denote significant differences at *P* < 0.05 from Student's *t*‐test.

Consistent with our previously published results (Anders *et al*., 2012; Lovegrove *et al*., 2013), TOT‐ and WE‐AX are decreased by approximately 50% in the TaGT43_2 and TaGT47_2 transgenic lines and by approximately 10% in the TaXAT1 line compared to control lines (Figure 2; Table S1). In the XE‐AX fraction, there is a similar decrease in AX content in the TaGT43_2 and TaGT47_2 lines to that in TOT‐AX and WE‐AX, but in the TaXAT1 line, AX content of this fraction is unaffected by the transgene. The amount of AX in the XU‐AX fraction is, however, largely unaffected by suppression of any of the three target biosynthetic genes (Figure 2). For the TaGT43_2 and TaGT47_2 transgenic lines, bound FA monomer content is decreased in the WE‐AX and XE‐AX fractions by similar amounts to AX content but bound FA dimer content is less affected (Figures 3 and 4). Therefore, the AX in these fractions of these transgenic lines appears to be more highly cross‐linked than in control lines. In contrast, the WE‐AX is much less feruloylated in the TaXAT1 transgenic lines than in the control line but largely unaffected in the XE‐AX fraction. The degree of feruloylation in the XU‐AX fraction is, like AX content, largely unaffected by suppression of any of the three target genes (Figure 3; Table S2), particularly expressed per unit AX (Figure 4).

Feruloylation of endosperm AX mostly occurs via esterified Ara*f* 3‐linked to mono‐substituted Xyl*p*, (Saulnier *et al*., 2007) although a smaller proportion may occur as feruloylated Ara*f* 3‐linked to di‐substituted Xyl*p* (Veličković *et al*., 2014). We therefore also examined the effect of the RNAi transgenes on the arabinosylation pattern in the WE‐AX and XE‐AX fractions by quantification of AX oligosaccharides (AXOS) by HPAEC and by proton NMR analyses. AXOS abundances present in the XE‐AX fraction or generated by subsequent endoxylanase digestion for the WE‐AX fraction are shown in Figure S1. For TaGT43_2 and TaGT47_2 RNAi lines, the AXOS are decreased more than the AX amount (Figure S1; compare with Figure 2a, b). This is because the substitution pattern is changed in these lines such that larger AXOS are released by GH11 digestion which are not identified by the HPAEC method (Lovegrove *et al*., 2013). In the TaXAT1 WE‐AX and XE‐AX fractions AXOS containing di‐substituted Xyl*p* show smaller decreases than other AXOS, with AXOS containing di‐substituted Xyl*p* actually increased relative to controls in XE‐AX (Fig. S1).

**Figure 5:**
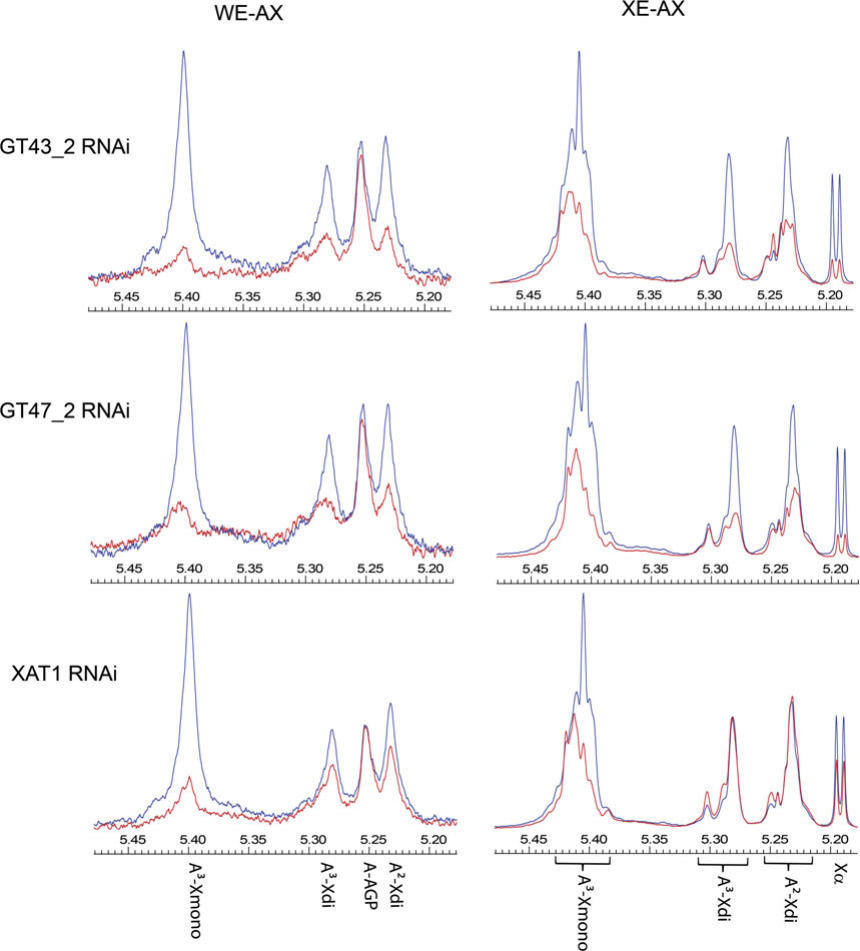
Representative 1H‐NMR spectra for transgenic (red) and azygous control (blue) samples; signal size is normalized to internal standard added to extract from a fixed dwt of endosperm. H1 resonances for Araf in AX are indicated: α‐(1,3)‐linked to mono‐substituted Xylp (A3‐Xmono), α‐(1,3)‐linked (A3‐Xdi), and α‐(1,2)‐linked (A2‐Xdi) to di‐substituted Xylp and for Araf in arabinogalactan peptide (A‐AGP; WE‐AX samples only). For XE‐AX samples, ranges are shown within which peaks for A3‐Xmono, A3‐Xdi and A2‐Xdi are known to occur from distinct oligosaccharides generated by GH11 digestion (Hoffman *et al*., 1991: Petersen *et al*., 2014); also a doublet from H1 α resonance for Xylp β‐(1,4)‐linked (Xα) is shown.

Interpretation of the HPAEC data, which is limited to analyses of AXOS with low DP, is complicated by the changed structure of the AX with fewer GH11 xylanase cleavage sites in the transgenic lines. NMR spectra of the region corresponding to the anomeric H1 resonances of the Ara*f* linked to the xylan backbone give an overview of all the Ara*f* 3‐linked to mono‐substituted Xyl*p* (A^3^‐Xmono), 3‐linked to di‐substituted Xyl*p* (A^3^‐Xdi) and 2‐linked to di‐substituted Xyl*p* (A^2^‐Xdi). Chemical shifts occur at around 5.40, 5.28 and 5.23 ppm, respectively, for A^3^‐Xmono, A^3^‐Xdi and A^2^‐Xdi in all AXOS arising from GH11 digestion, although the exact position varies according to substitutions of neighbouring Xyl*p* (Hoffmann *et al*., 1991; Petersen *et al*., 2014). Intact AX molecules give rise to broad peaks at these positions due to the multiplicity of contexts for the Ara*f* present (Petersen *et al*., 2014), whereas mixtures of specific AXOS from GH11 digestion give sets of overlapping but distinct peaks and this is seen here for the WE‐AX and XE‐AX fractions (Figure 5). For all RNAi lines, the NMR shows bigger decreases in both mono‐ and di‐substitution in WE‐AX than in XE‐AX; the effects are quantified by integration of peak areas for all spectra in Table 3. The TaXAT1 line shows far more decreased mono‐substitution than decreased di‐substitution; for the XE‐AX fraction, di‐substitution is actually increased by 30%–40% in TaXAT1 RNAi whilst mono‐substitution is decreased by 27% (Table 3). The increase in di‐substitution in XE‐AX explains why there is no overall decrease in Ara*f* content in TaXAT1 RNAi lines in this fraction (Figure 2).

**Table 3:** Estimates of Araf content with different linkages in mg/g dry weight flour from integration of peaks in H1‐NMR spectra, assuming peak area per ^1^H is the same as for the internal standard. Integrations were performed in regions A^3^‐X mono δ5.435–5.375, A^3^‐Xdi δ5.310–5.265, A^2^‐Xdi δ 5.255–5.210. For the region of A^2^‐Xdi peak in WE‐AX which overlaps with A‐AGP at around δ5.255 (Figure 6), area was estimated using values from the other side of the peak as the peak is symmetrical. Mean values are shown ± SEM, *n* = 3. H/A is ratio of transgenic mean to azygous control mean as %, with *P*‐value from Student's *t*‐test for H‐A comparison shown in parentheses

Fraction	RNAi target	Trans‐gene	A^3^‐Xmono		A^3^‐Xdi		A^2^‐Xdi	
			Mean	H/A	Mean	H/A	Mean	H/A
WE	TaGT43_2	A	0.37 ± 0.03	30% (0.002)	0.21 ± 0.01	62% (0.002)	0.23 ± 0.01	51% (0.000)
		H	0.11 ± 0.01		0.13 ± 0.01		0.12 ± 0.00	
	TaGT47_2	A	0.32 ± 0.03	30% (0.004)	0.18 ± 0.02	63% (0.014)	0.19 ± 0.00	57% (0.000)
		H	0.10 ± 0.01		0.12 ± 0.00		0.11 ± 0.01	
	TaXAT1	A	0.47 ± 0.02	29% (0.000)	0.24 ± 0.00	69% (0.000)	0.28 ± 0.00	69% (0.000)
		H	0.14 ± 0.01		0.17 ± 0.00		0.19 ± 0.00	
XE	TaGT43_2	A	4.21 ± 0.11	51% (0.000)	1.52 ± 0.04	53% (0.000)	1.84 ± 0.04	67% (0.000)
		H	2.15 ± 0.05		0.80 ± 0.01		1.23 ± 0.01	
	TaGT47_2	A	4.27 ± 0.10	58% (0.000)	1.47 ± 0.02	64% (0.000)	1.85 ± 0.03	72% (0.002)
		H	2.46 ± 0.12		0.94 ± 0.03		1.34 ± 0.07	
	TaXAT1	A	3.84 ± 0.12	73% (0.002)	1.25 ± 0.04	141% (0.000)	1.60 ± 0.05	130% (0.001)
		H	2.80 ± 0.05		1.76 ± 0.01		2.07 ± 0.04	

## Discussion

Our results show that in wheat RNAi lines with substantially decreased amount and chain length of endosperm AX (TaGT43_2 and TaGT47_2 RNAi lines) and altered AX structure (all RNAi lines), the magnitude of decreases in amounts of FA and particularly of diFA are much smaller or absent (Table 1). We assume, as is common, that all the bound FA and diFA in endosperm are ester‐linked to Ara*f* on AX which is supported by the lack of reports of ferulate ester‐linked to residues other than Ara*f* using approaches where the ester bonds are preserved on cell wall fractions from cereal grain (Saulnier *et al*., 1995) and grasses in general (Ishii, 1997; Mueller‐Harvey and Hartley, 1986). By separation of the endosperm AX into the WE‐AX, XE‐AX and XU‐AX fractions, we show that the smaller effects on FA and diFA than on AX are because the effects of the transgenes on amount of AX are greatest for the lowly feruloylated WE‐AX fraction, less for the moderately feruloylated XE‐AX and absent for the highly feruloylated XU‐AX fraction (Figures 2 and 3). However, the compositions of these fractions are not completely unaffected by the transgenes, at least for WE‐AX and XE‐AX. For TaGT43_2 and TaGT47_2 RNAi lines, diFA content per unit AX (Figure 4e, f) is increased although FA monomer (Figure 4a, b) is not. Thus, FA dimerization [diFA/(diFA + FA)] in WE‐AX is increased from 6% in azygous controls to 9% in TaGT43_2 and TaGT47_2 RNAi lines and in XE‐AX from 16% to 22%. For the TaXAT1 line, FA monomer and diFA content per unit AX are decreased to a similar degree in the WE fraction; hence, the degree of dimerization is unaffected by the transgene (6%) whereas in the XE fraction, there is a small but significant decrease in FA monomer per unit AX and no effect on diFA content resulting in an increase in dimerization from 16% in azygous controls to 19% in the homozygous line. Dimerization in the XU‐AX fraction is however unchanged at around 22% for all lines. Ultimately, the amount of dimerization that is possible depends on the fraction of FA monomer that are sufficiently close to oxidatively couple and it may be these are at a maximum for dimerization in the XU‐AX fractions.

The solubility of wheat grain AX is an important characteristic for end use as it is the WE‐AX that determines extract viscosity; a negative trait for nonfood uses of wheat, but desirable in food as soluble fibre. It has previously been suggested that the dominant characteristic determining AX solubility is amount of diFA (Saulnier *et al*., 2007). This is supported by the evidence here; AX from TaGT43_2 and TaGT47_2 RNAi lines has substantially shorter chain length (Freeman *et al*., 2016) and not much change in overall Ara*f*/Xyl*p* ratio (Figure 2h) so might be expected to have a higher proportion of AX in the WE‐AX fraction. In fact, this proportion is not much changed and this could be due to the overall greater amount of diFA per unit AX (Figure 4h).

We were also interested in the pattern of arabinosylation in the WE‐AX and XE‐AX fractions, especially since ferulate is believed to be exclusively linked to Ara*f* 3‐linked to Xyl*p* [since this is the only linkage found in feruloylated AX (Ishii, 1991; Lequart *et al*., 1999; Rhodes *et al*., 2002)]. HPAEC quantification of AXOS suggested that mono‐substitution and di‐substitution were equally decreased in TaGT43_2 and TaGT47_2 RNAi lines, whereas mono‐substitution was much more decreased than di‐substitution in TaXAT1 RNAi lines; the magnitude of decreases was greater in WE‐AX than XE‐AX fractions for all three RNAi lines (Figure S1). However, the AX structural changes in TaGT43_2 and TaGT47_2 RNAi lines also led to a shift to larger AXOS not detected by the HPAEC (Lovegrove *et al*., 2013). This problem is effectively overcome by NMR analysis, where A^3^‐Xmono, A^3^‐Xdi and A^2^‐Xdi signals are seen from all AXOS and whole AX chains at similar positions (Figure 5) with the exact position depending mostly on the substitution of immediately neighbouring Xyl*p* (Petersen *et al*., 2014). The NMR spectra confirm that in the TaGT43_2 and TaGT47_2 RNAi lines, there are decreases in the A^3^‐Xmono, A^3^‐Xdi and A^2^‐Xdi signals whereas in the TaXAT1 RNAi line, the decrease in A^3^‐Xmono signal is greater than for the A^3^‐Xdi and A^2^‐Xdi signals which are slightly decreased in WE‐AX and increased in XE‐AX (Table 3). In all NMR spectra for XE‐AX fractions, it is noticeable that some regions within the sets of peaks ascribed to A^3^‐Xmono, A^3^‐Xdi and A^2^‐Xdi are relatively unaffected by the transgenes even when the others are substantially decreased (Figure 5). AXOS where the neighbouring Xyl*p* is di‐substituted with Ara*f* are known to give rise to peaks in these regions [namely 5.42–5.44 for A^3^‐Xmono, 5.31 for A^3^‐Xdi and 5.25 for A^2^‐Xdi (Petersen *et al*., 2014)]. As A‐Xdi peak areas in Table 3 include these regions, they tend to be somewhat less affected than A^3^‐Xmono even for TaGT43_2 and TaGT47_2 RNAi lines. We know that AX deposited earlier in grain development is richer in di‐substitution (Pellny *et al*., 2012; Toole *et al*., 2010) and that RNAi transgenes driven by the HMW promoter tend to have more effect on cell wall polysaccharides later in development (Lovegrove *et al*., 2013; Nemeth *et al*., 2010). Therefore, these relatively unaffected regions may reflect AX rich in di‐substitution that is deposited too early in development to be affected by the RNAi suppression.

By contrast, feruloylation of AX appears to be maintained at a fairly constant level during development (Pellny *et al*., 2012) so a lack of effect on the degree of feruloylation of AX cannot be attributed to early deposited AX. In the XE‐AX fraction, there was no significant decrease in FA or diFA in the TaXAT1 RNAi line (Figure 3b, f; Table S2) despite the fact that there were substantial decreases in Xyl*p* mono‐substituted with 3‐linked Ara*f* to which this FA is linked, (around 40%) as indicated by NMR (Figure 5). In one model of feruloylation of AX, feruloylated Ara*f* is transferred onto the AX in the Golgi by the action of a glycosyl transferase (Buanafina, 2009; de Oliveira *et al*., 2015); the lack of effect of suppressing *TaXAT1* on feruloylation here indicates that TaXAT1 protein may only transfer nonferuloylated Ara*f*. This is compatible with the expression pattern of *TaXAT1* which is highest in starchy endosperm, a tissue with uniquely low levels of AX feruloylation (Pellny *et al*., 2012).

Our data show that FA and diFA content of starchy endosperm cell walls is substantially independent of the amount of AX when decreased by *TaGT43_2* and *TaGT47_2* suppression, and of the amount of Ara*f* 3‐linked to mono‐substituted Xyl*p* when decreased by *TaXAT1* suppression. The particular maintenance of diFA levels suggests that diFA content may be vital to the integrity of the endosperm cell walls. This is plausible because FA dimerization probably represents the dominant mode of covalent cross‐linking between chains in the cell walls of this tissue where 70% of the polysaccharide is AX and there is no lignin. When AX ferulate in wheat endosperm was decreased by heterologous expression of a fungal feruloyl esterase in another study, grain morphology was severely affected with an apparent collapse of endosperm tissue (Harholt *et al*., 2010). Cell wall integrity is sensed in higher plants, and compensatory changes occur in response to weakening, for example, by production of reactive oxygen species (ROS) to promote cell wall stiffening by increased peroxidative coupling (Voxeur and Höfte, 2016). Similar mechanisms could operate here and when the wall is weakened by the low AX content and shorter chains in the TaGT43_2 and TaGT47_2 lines, FA dimerization could be increased (as seen in WE‐AX and XE‐AX fractions) by ROS production and increased peroxidase activity, but greater feruloylation per unit AX may also be required as is observed overall (Figure 4d). This hypothesis could explain how the endosperm cell walls that are half as thick as normal in the TaGT43_2 and TaGT47_2 RNAi lines (Lovegrove *et al*., 2013) are able to maintain their form.

The wheat endosperm cell wall is a tractable system, and our results here may provide some general insight into AX feruloylation; however, it is a special case with simple, lowly feruloylated AX. Studies on the highly feruloylated GAX typical of the majority of grass tissue (e.g. in maize pericarp; Chateigner‐Boutin *et al*., 2016) are needed to establish the control of this key process in determining cell wall properties and grass biomass digestibility.

## Experimental procedures

### Plant material and growth conditions

Wild‐type wheat cv. Cadenza was grown in the field at Rothamsted Research farm. All other wheat *(Triticum aestivum* L. var. Cadenza) plants were grown in temperature‐controlled glasshouse rooms as previously described (Nemeth *et al*., 2010) in 25‐cm pots with five plants per pot. Within experiments, homozygous and azygous segregants descended from the same original transformant were grown at the same time in the same glasshouse room. All grains were harvested, and biological replicates consisted of grain from single pots and 5‐g grains were milled. Generation of transgenic lines is described in (Anders *et al*., 2012) for TaXAT1 RNAi lines and (Lovegrove *et al*., 2013) for TaGT43_2 and TaGT47_2 RNAi lines.

### Sequential extraction of fractions from white flour

White flour from wild‐type wheat cv. Cadenza was prepared using a Chopin CD1 mill (Calibre Control International Limited). All other white flour samples were prepared as previously described (Anders *et al*., 2012). For quantification of monosaccharide and phenolic acid content, fractions were prepared as represented in Figure 1. Alcohol insoluble residue (AIR) was prepared from 200 mg of white flour by extraction in 80% ethanol as previously described (Pellny *et al*., 2012) and dried by centrifugation under vacuum. The water‐extractable fraction (WE) was prepared by suspending the AIR in 1.5 mL water, mixing by rotation at 30 rpm for 30 min and centrifugation for 10 min at 10 000 *g*. Samples of supernatant (WE) were taken and dried as above. After washing with 1.5 mL water, the pellet was destarched by digestion at 80 °C for 30 min in 1 mL 0.1 m sodium acetate, pH5.2, 4 mm calcium chloride containing 250 units of α‐amylase (Sigma‐Aldrich, A3403), cooled to 50 °C before the addition of 10 μL pullulanase (Sigma‐Aldrich, P2986) and incubation at 50 °C for 30 min. Released glucose was removed by centrifugation, and the pellet washed twice with 1 mL water and dried as above. The xylanase‐extractable fraction (XE) was prepared by digestion with 4 μL xylanase GH11 (Prozomix, PRO‐E0062) and 1.9 μL lichenase (Prozomix, PRO‐E0017) in 1 mL water following the procedure described by Nemeth *et al*. (2010). The residue (xylanase‐unextractable fraction, XU) was washed with 1 mL water, dispersed in 1 mL water and dried as above. Samples for NMR analysis were prepared essentially as described above except that the WE fraction was digested with 0.5 units of α‐amylase in 4 mm calcium chloride for 15 min at 100 °C and arabinoxylan (AX) recovered by precipitation as described by Ordaz‐Ortiz and Saulnier (2005); the XE fraction was not destarched. For analysis of oligosaccharides (AXOS) released by digestion with xylanase (GH11) and lichenase in WE and XE fractions, AIR was prepared from 100 mg white flour as described by Saulnier *et al*. (2009), WE and XE fractions as for NMR analysis without drying the XE fraction, and then the WE fraction was digested with xylanase and lichenase as for the XE fraction except the total volume was 0.5 mL.

### Cell wall analyses of white flour and fractions sequentially extracted from white flour

#### Quantification of ferulic acid monomer (FA) and dehydrodimer (diFA) content

Cell wall bound phenolics were extracted from AIR prepared from white flour, or from WE, XE and XU fractions as previously described (Pellny *et al*., 2012).Quantification was by HPLC using a binary gradient with acetonitrile (solvent A) and 2% acetic acid (solvent B) either as described by Pellny *et al*. (2012) or essentially as described by (Pellny *et al*., 2012) except separating 20 μL of sample on a UPLC Kinetex Phenyl‐Hexyl (150 × 4.6 mm, 5 μm) column with the following gradient: linear 100% to 30% B, 0–12 min; isocratic 30% B, 12–14 min; Linear 30% to 100% B, 14 to 14.1 min; isocratic 100% B, 14.1 to 18 min and a flow rate of 2 mL/min. Quantification of FA was by integration of peak areas at 280 nm with reference to calibrations made using known amounts of pure compounds. Peaks of the major diFAs were identified by comparison with retention times and absorption spectra with pure standards kindly supplied by Professor John Ralph (Lu *et al*., 2012). Values reported for diFAs are the sum of amounts of 8‐5′ benzofuran (BF), 8‐O‐4′, 5‐5′ and 8‐5′ diFAs. Three further diFA standards (8‐8′, 8‐8′ aryltetralin and 8‐8′ tetrahydrofuran) were not detected in these samples. DiFA content of samples, relative to FA monomer, was calculated using areas of these peaks and response factors (relative response factors for 8‐5′BF, 8‐O‐4′, 5‐5′ and 8‐5′ diFAs were 0.501, 0.692, 0.417 and 0.456 respectively).

### Monosaccharide analysis by high‐performance anion‐exchange chromatography (HPAEC)

For total neutral sugar content of white flour, 10 mg of flour was finely ground using 3 × 3 mm diameter stainless steel ball bearings (Atlas Ball and Bearing Company Limited) and agitation for 1 min at 15 000 rpm in a Genogrinder (Spex Sample Prep^®^), dilution to 1 mg/mL and 200 μL aliquots (equivalent to 200 μg flour) dried by centrifugation under vacuum. For WE, XE and XU fractions, 37.5 μL, 12.5 μL and 50 μL aliquots (equivalent to extract from 5 mg, 2.5 mg and 10 mg white flour), respectively, were dried as above. Sugars were released by acid hydrolysis by dissolution in 400 μL 2 m trifluoroacetic acid and heating to 120 °C for 60 min and then dried as above and washed by dissolution in 500 μL water and drying. Calibration curves were generated by subjecting a range of amounts of commercially available sugars (Sigma‐Aldrich UK) to acid hydrolysis following the same protocol as for samples. Dried samples and standards were dissolved in 400 μL water and filtered through 0.45 μm PVDF disposable filters (Whatman) before separation by HPAEC (Dionex ICS‐5000+ HPIC, Thermo Scientific) on a CarboPac PA20 analytical column (3 × 150 mm) with a CarboPac PA20 guard column (3 × 30 mm) at 30 °C and equipped with an eluent generator with an EGC 500 KOH cartridge. The flow rate was 0.5 mL/min, and the gradient was isocratic 4.5 mm KOH, 0–13 min; linear 4.5–10 mm KOH, 13–14 min; linear 10–13 mm KOH, 14–15 min; linear 13–20 mm, 15–16 min; isocratic 20 mm 16–17 min; linear 20–4.5 mm KOH, 17–18 min followed by isocratic 4.5 mm KOH 18–23 min to equilibrate the column for the next injection. Detection was by electrochemical detector, and chromatograms were analysed and data calculated using Chromeleon 7.2 software (Thermo Scientific). AX content was calculated as the sum of arabinose and xylose except in WE‐AX fractions and flour which were corrected for arabinogalactan content assuming an arabinose to galactose ratio of 0.7 (Ordaz‐Ortiz and Saulnier, 2005).

### Analysis of arabinoxylan (AX) and (1‐3):(1‐4)‐β‐D‐glucan content by HPAEC

AXOS and glucans were separated and chromatograms analysed as described by (Kosik *et al*., 2017). Integrated peak areas were recorded for each AXOS and for G3 and G4 peaks derived from (1‐3):(1‐4)‐β‐D‐glucan and the ratio of peak area in transgenic lines to that in corresponding azygous control lines calculated.

### ^1^H‐NMR analysis

Samples were suspended in D_2_O (1 mL) containing 0.05% (wt/vol) d_4_‐trimethylsilyl propionic acid. ^1^H‐NMR spectra were recorded at 300 K on a Bruker Avance NMR spectrometer (Bruker Biospin), operating at 600.05 MHz, equipped with a selective inverse probe. Spectra were collected using 128 scans using a zgpr pulse sequence with a 90° pulse angle. The residual water signal was suppressed by presaturation during a 5‐s delay. Spectra consisted of 64‐K data points over a sweep width of 12 ppm. Free induction decays were Fourier transformed using an exponential window with a line broadening of 0.5 Hz. Phasing and baseline correction were carried out automatically within the instrument's TOPSPIN v. 2.1 software. AX peak assignments were based on (Anders *et al*., 2012), and the H1 Araf peak assignment of arabinogalactan peptide was made by comparison with an authentic standard.

## Supporting information


**Figure S1** AXOS abundance determined by HPAEC in white flour fractions from transgenic wheat lines.
**Table S1** Neutral sugar content of white flour fractions from transgenic wheat lines.
**Table S2** Ferulate dehydrodimer composition of white flour fractions from wild‐type wheat and transgenic wheat lines plus control lines.
