# Supplementary material for: Feruloylation and structure of arabinoxylan in wheat endosperm cell walls from RNAi lines with suppression of genes responsible for backbone synthesis and decoration
Source: Plant Biotechnol J. 2017 Apr 21;15(11):1429–38. doi: 10.1111/pbi.12727 (PMC5633762; doi:10.1111/pbi.12727)
Supplement: Supplementary file 1 — Figure S1 AXOS abundance determined by HPAEC in white flour fractions from transgenic wheat lines. Table S1 Neutral sugar content of white flour fractions from transgenic wheat lines. Table S2 Ferulate dehydrodimer composition of white flour fractions from wild‐type wheat and transgenic wheat lines plus control lines. [file PBI-15-1429-s001.docx]

**Table S1.** Neutral sugar content of white flour, and fractions sequentially extracted from white flour from transgenic wheat lines homozygous (H) for RNAi constructs suppressing AX biosynthetic genes, and azygous (A) control lines. Contents of all fractions are expressed as mg.g^-1^ dry weight flour. WE = water-extractable fraction; XE = xylanase extractable fraction solubilised by digestion with GH11 endoxylanse and lichenase; XU = xylanase-unextractable fraction. Values are average ± SEM, n=3 or 4 replicates except TaXAT1-2 H TOT where n=2.

| Fraction | Sugar | Line | | | | | |
| --- | --- | --- | --- | --- | --- | --- | --- |
|  |  | TaGT43_2-3 | | TaGT47_2-4 | | TaXAT1-2 | |
|  |  | A | H | A | H | A | H |
| WE | Arabinose | 2.7 ± 0.0 | 2.3 ± 0.0 | 2.6 ± 0.0 | 2.2 ± 0.0 | 2.8 ± 0.0 | 2.4 ± 0.1 |
|  | Xylose | 2.8 ± 0.0 | 1.5 ± 0.0 | 2.8 ± 0.0 | 1.3 ± 0.0 | 2.6 ± 0.0 | 2.3 ± 0.1 |
|  | Galactose | 2.4 ± 0.0 | 2.5 ± 0.0 | 2.3 ± 0.0 | 2.4 ± 0.0 | 2.1 ± 0.0 | 2.3 ± 0.1 |
|  | Glucose | 5.6 ± 0.1 | 5.4 ± 0.1 | 5.5 ± 0.1 | 4.9 ± 0.1 | 6.0 ± 0.0 | 5.5 ± 0.3 |
|  | Mannose | trace | trace | trace | trace | trace | trace |
| XE | Arabinose | 4.5 ± 0.1 | 2.5 ± 0.0 | 4.5 ± 0.0 | 2.3 ± 0.2 | 4.0 ± 0.3 | 4.1 ± 0.0 |
|  | Xylose | 5.4 ± 0.1 | 3.9 ± 0.0 | 5.4 ± 0.1 | 3.1 ± 0.3 | 5.4 ± 0.2 | 5.6 ± 0.0 |
|  | Galactose | trace | trace | trace | trace | trace | trace |
|  | Glucose | 3.8 ± 0.3 | 4.3 ± 0.1 | 3.5 ± 0.1 | 2.4 ± 0.2 | 3.8 ± 0.3 | 3.1 ± 0.2 |
|  | Mannose | ND | ND | ND | ND | ND | ND |
| XU | Arabinose | 1.3 ± 0.0 | 1.2 ± 0.0 | 1.4 ± 0.0 | 1.2 ± 0.0 | 1.3 ± 0.0 | 1.2 ± 0.0 |
|  | Xylose | 1.3 ± 0.0 | 1.2 ± 0.0 | 1.3 ± 0.0 | 1.1 ± 0.0 | 1.3 ± 0.0 | 1.2 ± 0.1 |
|  | Galactose | 0.5 ± 0.0 | 0.5 ± 0.0 | 0.5 ± 0.0 | 0.5 ± 0.0 | 0.6 ± 0.0 | 0.5 ± 0.0 |
|  | Glucose | 3.3 ± 0.1 | 3.2 ± 0.1 | 3.1 ± 0.0 | 3.4 ± 0.1 | 3.1 ± 0.1 | 3.0 ± 0.1 |
|  | Mannose | 0.4 ± 0.0 | 0.5 ± 0.0 | 0.5 ± 0.0 | 0.6 ± 0.0 | 0.5 ± 0.0 | 0.3 ± 0.0 |
| TOT | Arabinose | 11.9 ± 0.8 | 7.8 ± 0.4 | 12.2 ± 0.7 | 7.7 ± 0.2 | 12.2 ± 0.7 | 11.9 ± 0.2 |
|  | Xylose | 17.3 ± 0.9 | 8.2 ± 0.7 | 17.6 ± 0.7 | 8.0 ± 0.5 | 19.0 ± 1.5 | 16.4 ± 0.3 |
|  | Galactose | 5.6 ± 0.2 | 5.6 ± 0.2 | 5.6 ± 0.2 | 5.4 ± 0.1 | 5.7 ± 0.4 | 6.2 ± 0.1 |
|  | Glucose | 773 ± 36 | 771 ± 60 | 805 ± 30 | 755 ± 38 | 854 ± 63 | 880 ± 26 |
|  | Mannose | 1.0 ± 0.1 | 1.2 ± 0.1 | 1.3 ± 0.1 | 1.3 ± 0.1 | 1.5 ± 0.1 | 1.4 ± 0.1 |

**Table S2.** Content of the four diferulates (diFA) detected in white flour, and fractions sequentially extracted from white flour from wheat cv Cadenza and transgenic wheat lines homozygous (H) for RNAi constructs suppressing AX biosynthetic genes, and azygous (A) control lines. WE = water-extractable fraction; XE = xylanase extractable fraction solubilised by digestion with GH11 endoxylanse and lichenase; XU = xylanase-unextractable fraction. (ND = not detected; BF = benzofuran). Values are average ± SEM; for WE, XE and XU n=4 replicate sequential extractions for TOT n=3.

| Fraction | Line |  | diFA µg.g-1 dwt | | | |
| --- | --- | --- | --- | --- | --- | --- |
|  |  |  | 8-5' | 5-5' | 8-O-4' | 8-5' BF |
| WE | Cadenza | WT | ND | ND | 0.3 ± 0.01 | 0.7 ± 0.05 |
|  | TaGT43_2 | A | ND | ND | 0.2 ± 0.01 | 0.4 ± 0.01 |
|  |  | H | ND | ND | 0.2 ± 0.00 | 0.3 ± 0.01 |
|  | TaGT47_2 | A | ND | ND | 0.1 ± 0.01 | 0.4 ± 0.01 |
|  |  | H | ND | ND | 0.1 ± 0.01 | 0.3 ± 0.00 |
|  | TaGT61_1 | A | ND | ND | 0.5 ± 0.00 | 0.7 ± 0.01 |
|  |  | H | ND | ND | 0.1 ± 0.01 | 0.3 ± 0.01 |
| XE | Cadenza | WT | 1.4 ± 0.14 | 1.7 ± 0.29 | 2.1 ± 0.08 | 2.8 ± 0.10 |
|  | TaGT43_2 | A | 3.1 ± 0.13 | 2.6 ± 0.07 | 2.3 ± 0.10 | 3.4 ± 0.16 |
|  |  | H | 3.1 ± 0.04 | 2.2 ± 0.02 | 2.5 ± 0.06 | 2.9 ± 0.05 |
|  | TaGT47_2 | A | 3.1 ± 0.13 | 2.8 ± 0.10 | 2.6 ± 0.09 | 3.2 ± 0.10 |
|  |  | H | 2.3 ± 0.08 | 1.9 ± 0.04 | 1.9 ± 0.06 | 2.1 ± 0.07 |
|  | TaGT61_1 | A | 3.2 ± 0.13 | 2.7 ± 0.07 | 2.8 ± 0.12 | 3.1 ± 0.11 |
|  |  | H | 3.7 ± 0.13 | 3.0 ± 0.06 | 2.7 ± 0.08 | 3.5 ± 0.10 |
| XU | Cadenza | WT | 1.3 ± 0.03 | 2.7 ± 0.06 | 3.2 ± 0.07 | 1.6 ± 0.02 |
|  | TaGT43_2 | A | 2.0 ± 0.04 | 2.6 ± 0.04 | 3.9 ± 0.08 | 2.5 ± 0.03 |
|  |  | H | 1.7 ± 0.02 | 2.5 ± 0.04 | 3.5 ± 0.08 | 2.2 ± 0.02 |
|  | TaGT47_2 | A | 2.6 ± 0.05 | 3.2 ± 0.04 | 4.6 ± 0.11 | 3.0 ± 0.07 |
|  |  | H | 2.1 ± 0.03 | 2.4 ± 0.02 | 3.6 ± 0.04 | 2.3 ± 0.03 |
|  | TaGT61_1 | A | 2.5 ± 0.07 | 3.0 ± 0.03 | 4.2 ± 0.06 | 3.0 ± 0.04 |
|  |  | H | 2.3 ± 0.07 | 2.8 ± 0.04 | 4.1 ± 0.05 | 2.5 ± 0.03 |
| TOT | Cadenza | WT | 4.2 ± 0.21 | 5.5 ± 0.12 | 4.9 ± 0.13 | 6.5 ± 0.16 |
|  | TaGT43_2 | A | 4.9 ± 0.10 | 4.6 ± 0.07 | 5.7 ± 0.12 | 6.7 ± 0.19 |
|  |  | H | 3.6 ± 0.01 | 3.3 ± 0.08 | 4.9 ± 0.07 | 5.5 ± 0.15 |
|  | TaGT47_2 | A | 5.7 ± 0.17 | 5.2 ± 0.22 | 6.8 ± 0.21 | 7.0 ± 0.05 |
|  |  | H | 3.8 ± 0.16 | 3.4 ± 0.18 | 5.1 ± 0.44 | 5.4 ± 0.12 |
|  | TaGT61_1 | A | 6.1 ± 0.15 | 5.1 ± 0.16 | 7.4 ± 0.43 | 7.8 ± 0.12 |
|  |  | H | 5.6 ± 0.08 | 5.3 ± 0.05 | 6.7 ± 0.20 | 7.1 ± 0.03 |


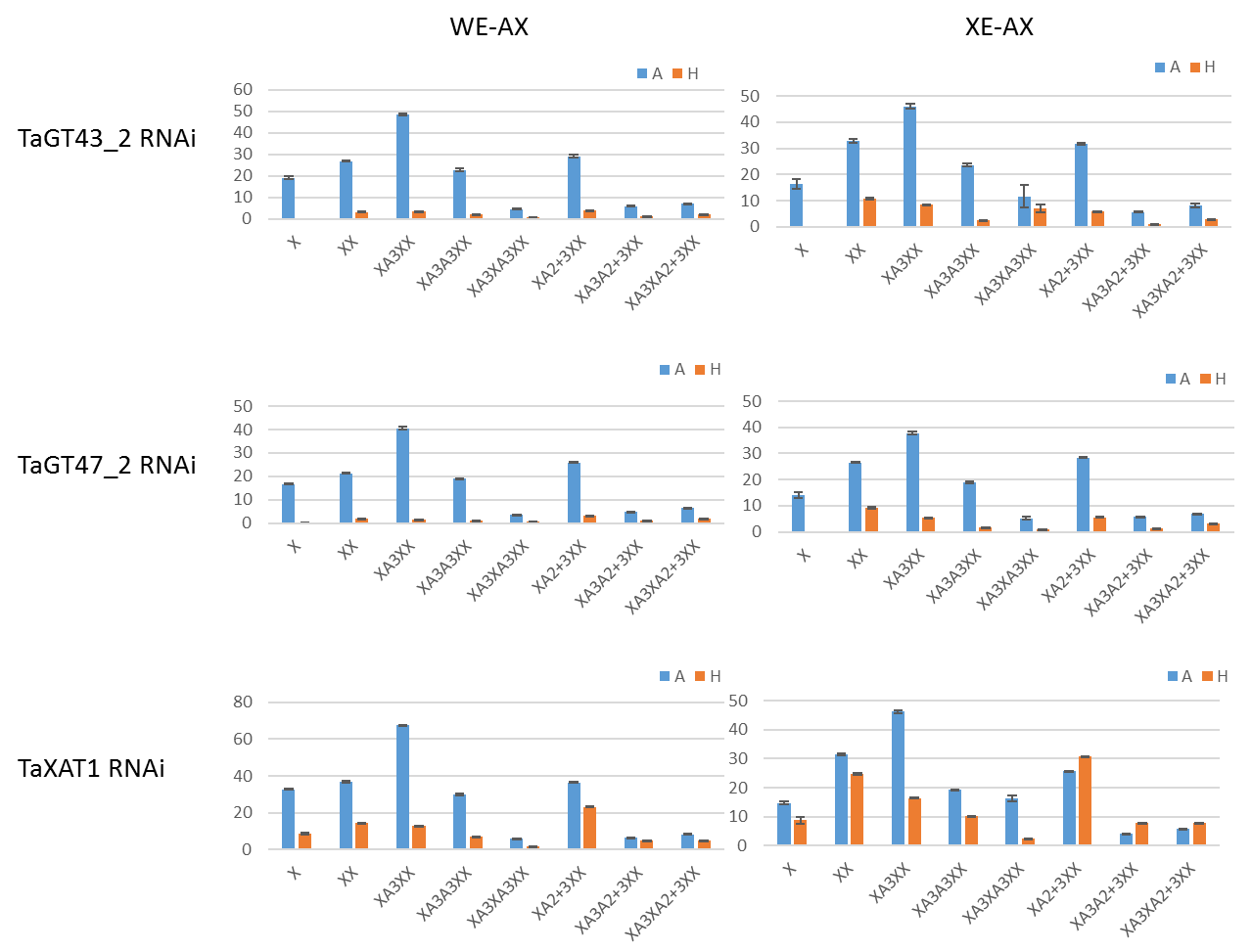


**Figure S1.** Abundance (from HPAEC peak area) of the major oligosaccharides resulting from digestion with GH11 endo-xylanase and lichenase of WE-AX and XE-AX fractions sequentially extracted from white flour of transgenic wheat lines homozygous for RNAi constructs (H) or azygous controls (A). XA3XX, XA3A3XX and XA3XA3XX are oligosaccharides containing only mono-substituted xylose residues; the XA2+3XX oligosaccharide contains only di-substituted xylose residues and XA3A2+3XX and XA3XA2+3XX contains both mono- and di-substituted xylose residues. For structures of oligosaccharides see Anders et al. (2012). Values are average ± SEM, n=3.
